# The Mediterranean Athlete’s Nutrition: Are Protein Supplements Necessary?

**DOI:** 10.3390/nu12123681

**Published:** 2020-11-29

**Authors:** Catherine L. Passariello, Silvia Marchionni, Mariateresa Carcuro, Giorgia Casali, Alberto della Pasqua, Silvana Hrelia, Marco Malaguti, Antonello Lorenzini

**Affiliations:** 1 Independent Researcher, Miami, FL 33101, USA; c.l.passariello@gmail.com; 2 Department of Biomedical and Neuromotor Sciences, University of Bologna, 40126 Bologna, BO, Italy; silvia.marchionni@unibo.it (S.M.); antonello.lorenzini@unibo.it (A.L.); 3 Studio Medico, 40024 Castel San Pietro Terme, BO, Italy; mariateresa.carcuro@gmail.com; 4 Independent Researcher, 47122 Forlì, FC, Italy; giorgia.casali1995@hotmail.it; 5 Poliambulatorio Obiettivo Benessere, 47030 San Mauro Pascoli, FC, Italy; albydelpas@virgilio.it; 6 Department for Life Quality Studies, University of Bologna, 47921 Rimini, RN, Italy; silvana.hrelia@unibo.it

**Keywords:** endurance, lifting, diet, macronutrients, protein, supplements

## Abstract

(1) Background: It is recommended that an athlete, in order to ensure correct nutrition and performance, should consume between 1.2 and 2.0 g/kg/day of protein, while the daily recommended protein intake for a non-athlete is 0.8and 0.9 mg/kg/day. It is unclear if athletes living in Mediterranean countries are able to meet protein requirements without supplementation, since Mediterranean diet de-emphasizes meat and meat products. (2) Methods: 166 athletes (125 males) enrolled between 2017 and 2019 were required to keep a dietary journal for three consecutive days (2 workdays and 1 weekend day). Athletes had to be >18 years old, train in a particular sport activity more than 3 h a week and compete at least at an amateur level. Journal data were collected and then translated into macro-nutrient content (grams of protein, carbohydrates, and lipids) by a nutritionist. (3) Results: The protein intake reported by this specific population vary slightly from the Academy of Nutrition and Dietetics (AND), Dietitians of Canada (DC), and the American College of Sports Medicine (ACSM) joint statement recommendation level. Average protein levels without protein supplementation fell within the protein guidelines. Counterintuitively, the intake among those who supplemented their diet with protein was higher compared with those who did not, even when excluding the contribution of supplements. Although the majority of subjects participating in the study were able to meet protein intake recommended for athletes without protein supplementation, 27% of athletes were below the guideline range. (4) Conclusions: these data suggest that athletes’ nutrition should be more often evaluated by a nutritionist and that they will benefit from increasing their nutritional knowledge in order to make better food choices, resorting to protein supplementation only when effectively needed.

## 1. Introduction

Many factors are key to design the optimal nutrition for athletes, total caloric intake, nutrient timing and periodization, macro- and micronutrient intake [1]. Here, we are addressing macronutrient needs of Mediterranean athletes with a focus on total daily protein intake. There seems to be a cultural shift in the dietary realm in which there is a marked increase on the importance of protein intake. Historically, fat intake was deemed as the cause of obesity and cardiovascular diseases, while now the finger is being pointed at carbohydrates, most likely related to the diabetes incidence in developing countries. There is also a parallel increase in protein supplement companies ultimately leading to marketing campaigns targeting also non-athletes that focus on the necessity of dietary integration without providing warnings on the possible risks from diets too high in protein [2].

The US Dietary Reference Intakes (DRI) specify a daily dietary protein intake for all individuals aged 19 years and older of 0.8 g/kg/day (Institute of Medicine, 2005). More specific to athletes, in 2016 the Academy of Nutrition and Dietetics (AND), Dietitians of Canada (DC), and the American College of Sports Medicine (ACSM) published their joint stance on the correct nutrition supporting athletic performance. They stated that protein ingestion level for athletes should fall between 1.2 and 2.0 g/kg/day, this range being necessary to support metabolic adaptation, repair, remodeling, and for protein turnover [3]. In contrast with previous published recommendations [4,5], this joint stance does not distinguish specific protein recommendation based on sport types or gender. The importance of protein intake for athletes is to preserve the lean body mass. Data suggest that protein intake between 1.8 and 2.4 g/kg/day are necessary to preserve lean body mass in individuals who are in caloric restriction, secondary to either dietary restriction or high caloric expenditure [6]. Moreover, during a heavy resistance training program lasting 8 weeks, Antonio et al. [7] observed positive body composition remodelling by applying high protein intake, up to 3.4 g/kg/day.

Increase in protein recommendations for athletes seems to follow the socio-cultural trend “carbs bad/protein good” as well as the dictates of the Paleo or Carnivore diets [8,9,10]. There is not a good grasp, however, of the athlete’s ability to meet the daily recommended protein intake while living in countries that have a tradition of following the Mediterranean diet. Although not strictly defined, the Mediterranean diet is the traditional diet that is eaten by populations surrounding the Mediterranean Sea. It is based on a high intake of fruits, vegetables, legumes, and unrefined cereals, a moderate to high intake of olive oil and fish (depending on the proximity to the sea), a moderate intake of milk, yogurt, and cheese, and a low consumption of meat or meat products [11]. A recent review of the literature sought to more clearly define the Mediterranean diet and concluded that it is a diet that provides 36.6 ± 4.9% energy from total fat, 42.8 ± 3.3% energy from carbohydrate and 14.9 ± 2.3% from protein [12]. Although proteins are found in other foods (grains, legumes, and milk products), meat and fish have the highest concentration of high-quality protein. People following the Mediterranean diet eat a maximum of one serving per week of red meat while a general Western diet provides up to three servings per week [13,14]. Protein intake is crucial in an athlete’s performance as it is necessary for the physiological remodeling that is desired from training, i.e., increase in lean muscle mass accompanied by a loss of fat mass [6]. The Western diet allows an athlete to easily reach adequate protein intake as demonstrated by previous studies [15]. However, the Western diet is not considered an optimal dietary approach to meet micronutrient and antioxidants needs [16]. On the contrary, the Mediterranean diet is a healthy dietary approach also for athletes, but its ability to provide sufficient proteins to athletes is still not clear. Monitoring protein intake in athletes living in Mediterranean countries allows the evaluation of protein supplementation as necessary or only just hype. In order to assess the athlete’s ability to reach the minimum suggested protein levels in a culture that predominantly follows the Mediterranean diet, we surveyed a wide range of athletes that at least competed at an amateur level, resided in Italy or Spain, and tracked their eating and sport practicing habits for three consecutive days, differentiating food from supplement-originating macronutrients.

## 2. Materials and Methods

Between 2017 and 2019, Exercise and Sport Science students from the University of Bologna, set to recruit amateur athletes willing to agree to journal their food intake for three consecutive days (2 workdays and 1 weekend day). The study was carried out according to the Declaration of Helsinki. The diaries were filled out anonymously, and the nutritionist used in the analysis was blinded to the participants as she was not involved in the recruitment of the subjects. Our Bioethics Committee does not assign a protocol number to completely anonymous surveys such as this one. Study participants had to be greater than 18 years old, at least competeing at amateur level, and practice at least 3 h/week in one sport, and reside in Italy or Spain at the time of the study participation. Protein supplementation was captured but was not an inclusion or exclusion criteria. If subjects met criteria, they were asked to sign a consent form, at which points participants were explained in detail how to fill-out a 3-day food diary, an instrument commonly used to access food intake [17,18] and could ask any clarifying questions (3-day food diary facsimile is available as Supplementary Materials). A total of 166 participants completed the dietary journal, 125 males and 41 females. Subjects were grouped by metabolic activity type: anaerobic, aerobic, and mixed exercise according to the metabolic features of the sport practiced.

The diary was designed by a nutritionist, set to capture food intake description, quantity, and time of meal/snack, as well activity description (type and duration). Athletes were asked to track all intake, whether liquid or solid and whether from foods or supplements. Journal data were then translated into macronutrient content (protein, carbohydrates, and lipids (g)) by the same nutritionist for every journal. The food database provided by the Council for Agricultural Research and Economics—Research Center on Food and Nutrition (CREA AN), that holds a total of 790 different foods and their nutritional characteristics (macronutrient content for every 100 g) was used to translate diary information into macronutrient content [19]. The de-identified information was entered into an excel file (Microsoft Corporation, Redmond, WA, USA) and then transferred to GraphPad Prism version 7.00 (GraphPad Software, San Diego, CA. USA) for statistical analysis.

The data were checked for normal distribution with the use of the D’Agostino and Pearson test (*p* > 0.05). Since the data passed the normality test, parametric statistics were then used. In order to test if our athletic population was able to intake the recommended protein amounts we used the one-way *t* test setting the hypothetical mean at 1.6 g/kg/day and significance threshold set at *p* < 0.05. The hypothetical mean of adequate protein intake of 1.6 g/kg/day was obtained by calculating the mean of the range provided by the joint statement from AND, DC and ACSM (1.2–2.0 mg/kg/day). The data were then subdivided by sport type, and a one-way *t* test was used to see if there were any differences in protein intake by sport type versus the hypothetical mean. When controlling for repeated measures, Sidak’s multiple comparisons test was used to test for a treatment effect of protein supplementation in subjects who chose to supplement their diet, following a significantly different two-way ANOVA result.

Tukey’s post hoc test was used following other ANOVA results for comparing the six available groups.

## 3. Results

Recruited participants were predominantly of Caucasian descent and Italian in nationality; only cross fit athletes were prevalently Spanish citizens. Anthropometric data of study participants (subdivided by sport type and gender) are reported in Table 1. Listed below are the sports that were included in each sport sub-type.

- Aerobic: triathlon, long distance running, and cycling;
- Anaerobic: body building and sprint swimming;
- Mixed: soccer, ultimate frisbee, swimming, volleyball, and cross-fit, rugby, basketball and karate.

As expected, females weighed significantly less than their male counterparts in all sport types, F (5, 160) = 22.92, *p* < 0.0001. Using a two-way ANOVA test, we found that body mass index (BMI) was significantly different between the six group (F (5, 160) = 9.233, *p* < 0.0001). Tukey’s test for multiple comparisons revealed that the only significant gender difference in BMI found among the three sport type groups was between the males and females that were training in anaerobic sports (23.5 vs. 20.7 kg/m^2^, *p* = 0.034). In addition, two-way ANOVA showed no gender dependent difference in training time (hours/week) (F (1, 160) = 0.2753, *p* = 0.6005, Supplemental Figure S1).

### 3.1. Percent Macronutrients

The percent of macronutrients consumed as part of their daily dietary intake (excluding supplement) was calculated (Table 2). When compared to the macronutrient definition of Mediterranean Diet as described by Davis, 2015, [12] some important differences were noticeable: across sport type, they consumed more carbohydrates (47.6 ± 8.8%vs 42.8 ± 3.3%), more protein (17.5 ± 4.3% vs. 14.9 ± 2.3%) and less fat (29.8 ± 7.1% vs. 36.6 ± 4.9%). Percent carbohydrate and percent lipid intake was independent of sport type and gender (supplemental Figure S2). Statistics did, however, reveal a statistically significant different between males in females in the anaerobic sport group (Supplemental Figure S2). The mean protein dietary intake (excluding protein obtained from supplements) for all the athletes was 1.48 ± 0.46 g/kg/day, and was significantly different from the hypothetical mean of 1.6 g/kg/day (*p* < 0.001). Suggesting that the athletic population surveyed was not meeting the recommended protein daily intake. Following, the surveyed population was then subdivided by the prevalent metabolic components of their discipline and the same analysis was completed in hopes to discover any differences by sport type. Both the anaerobic and mixed sports groups were statistically different from the hypothetical mean of 1.6 g/kg/day. Athletes who perform an aerobic sport had a higher protein intake than the other two sub-types and resulting in a non-statistical difference from the hypothetical mean. Statistical analysis also showed no significant gender-dependent variation in protein intake in the total group as well as the sport type sub-groups (Supplemental Figure S3).

### 3.2. Comparison between Athletes Who Use and Do Not Use Protein Supplements

**Figure 1:**
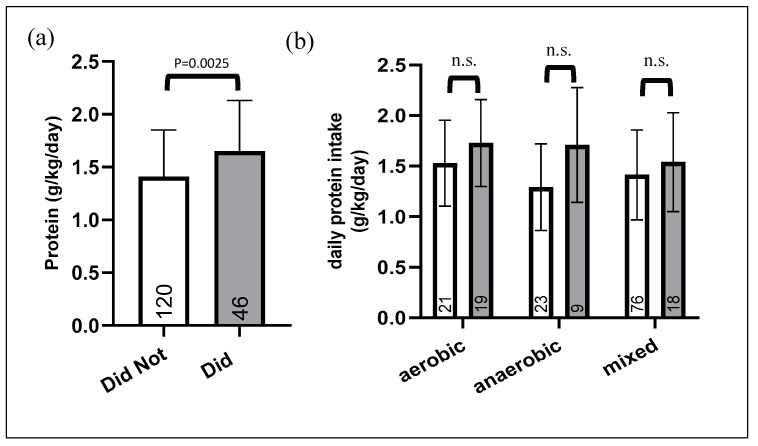
Daily protein intake from food-only among those who supplement and those who do not. (**a**) Bar graph of protein intake (excluding supplements) subdivided by those who did not supplement and those who did. Numbers inside the bars represent number of subjects. (**b**) Graphical representation of the effect of sport type and of an athlete’s choice to take protein supplements on daily protein intake. White bars are the athletes who did not take supplements. Gray bar are the athletes who did chose to take supplements, but data is excluding protein from supplements. Numbers inside the bars represent number of subjects. Two-way ANOVA showed a population dependent difference (F (1, 160) = 8.454, *p* = 0.0042), meaning that individuals who decided to take supplements were statistically different from those who did not take supplements in their daily protein intake. Sidak’s multiple comparisons test did not show any significant differences in each sport group, n.s. = not significant.

An unpaired *t*-test revealed that athletes who chose to supplement had a significantly higher protein intake than those who did not supplement, when only considering protein from food intake (1.64 ± 0.48 vs. 1.41 ± 0.44 g/kg/day) (Figure 1a). A two-way ANOVA followed by Sidak’s multiple comparisons showed that protein intake was dependent on supplementation habits (F (1, 160) = 8.454, *p* = 0.0042); however, the post hoc analysis only detected a trend in the anaerobic group (*p* = 0.057) (Figure 1b). As Figure 1 demonstrates, those who did not supplement their diet had a lower daily protein intake regardless of sport type.

When these groups were then checked to see if their mean was significantly different from the hypothetical mean of 1.6 g/k/day, by a one-way *t* test, we found that the anaerobic and mixed sport athletes means were significantly lower than the hypothetical mean in those subjects that chose not to take protein supplements. While in contrast those who chose to supplement had a mean that was not significantly different from the hypothetical mean, suggesting a sufficient protein intake even prior to supplementation.

In efforts to further understand the differences between the population who choose to supplement and those who do not, we looked to see what percentage of each group was below the minimum recommendation of 1.2 g/kg/day of daily protein intake (Supplemental Table S1). Although the majority of subjects participating in the study were able to meet protein intake recommended for athletes without protein supplementation, 27% of athletes were below the guideline range. There was a higher percentage of athletes across all sport types that were below the 1.2 g/kg/day threshold in those groups who did not supplement their diet. This is another manner to corroborate our previous observation, i.e., that athletes who supplement are not necessarily the ones who are in need. Additionally, when looking at the group who did supplement, individuals were below the 1.2 g/kg/day threshold, and after supplementing, only two of those individuals took enough supplements to be in range. This is yet another indication that supplementation is occurring in these athletes haphazardly.

### 3.3. Athletes Using Protein Supplements

**Figure 2:**
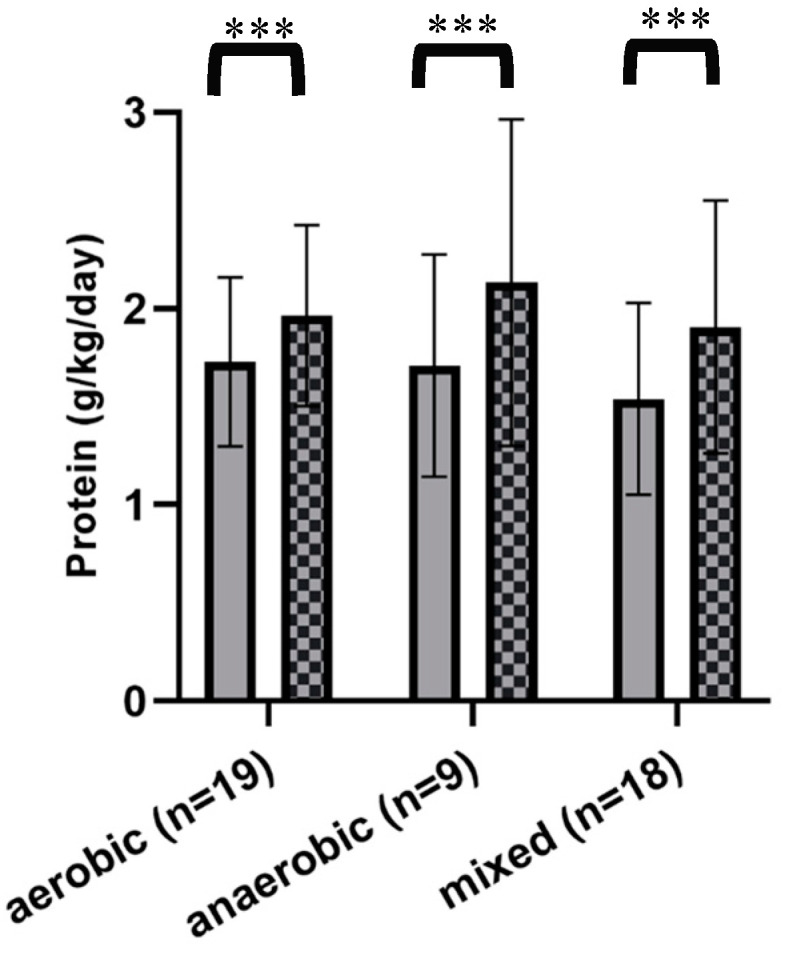
Graphical representation of the effect of protein supplementation for athletes who choose to supplement their diet with protein. Gray bars represent the daily protein intake excluding supplements, checkered bars represent matched individuals’ intake including protein from supplements. Two-way ANOVA repeated measures showed a significant effect of supplementation (F (1, 43) = 79.43, *p* < 0.0001), and Sidak’s multiple comparisons test showed that supplementation was effective in all sport groups, *** = *p* < 0.001.

There were only 28% of all participants (46/166) who reported taking protein dietary supplements (Figure 1a). When subdivided by sport type (Table 3), athletes who practice an aerobic sport supplemented their diet at a higher rate than the other two sport types; 47.5% of aerobic athletes, 28% of anaerobic athletes, and 19% of mixed athletes supplemented their diet. Figure 2 reports protein daily intake including and excluding protein grams attributed to supplements of athletes consuming supplements. A two-way ANOVA repeated measures test was used to determine if protein supplementation was an effective intervention to increase an athlete’s daily protein intake. Two-way ANOVA showed a significant effect of supplementation (F (1, 43) = 79.43, *p* < 0.0001), and Sidak’s multiple comparisons test showed that supplementation was effective in all sport groups, *** = *p* < 0.001.

### 3.4. Relationship between Protein Intake and Weekly Training Hours

**Figure 3:**
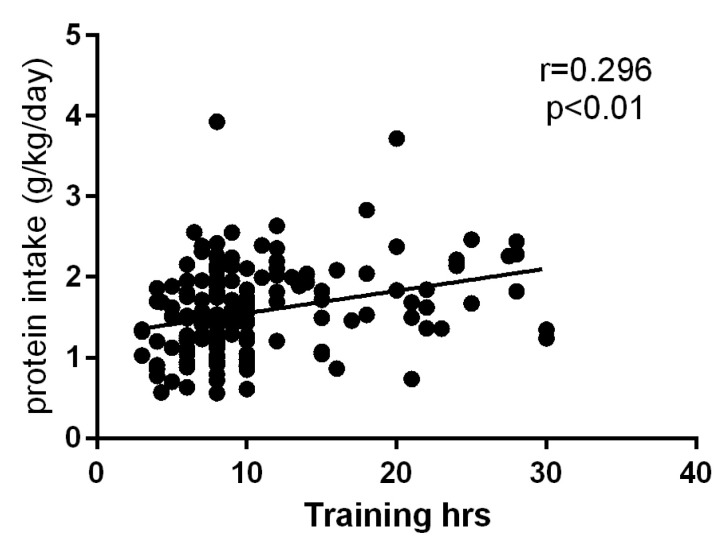
Relationship between total protein intake (including supplements) and weekly training hours. Correlation revealed a significant positive relationship between the two variables (*p* < 0.01).

Finally, we looked to see if there was a relationship between total protein intake including supplement and weekly training hours. Statistical analysis revealed a significant, but weak relation between the two variables (Figure 3). Similarly, even when analyzed by sport type this relationship was weak but significant (Supplemental Figure S4).

## 4. Discussion

In this study, we evaluate if the Mediterranean diet, that is recommended among the general population to promote health [20,21,22], may provide the protein quantity necessary for optimal athletic performance. Indeed, the mean of protein daily intake for all study participants was 1.48 ± 0.46 g/kg/day, an amount within the AND, DC and ACSM joint statement range of recommended intake (1.2–2.0 g/kg/day), but significantly below its mean. Our results acquire even more significance considering the findings of a recently published systematic review by Jenner SL et al. [23], who demonstrated that among professional and semiprofessional athletes protein intake is often above the upper limit of the recommended range. A relevant observation is that in our group, 27 percent (*n* = 44) of the athletes fell below the minimum amount of protein intake of 1.2 g/kg/day. This percentage is rather large considering our population is comprised of competitive athletes.

Our survey data showed that the recruited athletes consumed macronutrients consistent with the Mediterranean diet as described by Davis et al. and Feart et al. [12,24]. Italy being a country with a moderate-to-high degree of Mediterranean diet adherence [25], it was expected that the athletes surveyed follow the Mediterranean Diet, but there was a slight variation in which the athletes sacrificed fat intake with protein. A general basic knowledge of the increase protein requirement in sport nutrition by these athletes was probably what permitted this small adjustment.

Although in the past, expert opinions and guidelines suggested that different sport activities required different protein intake [4,5], more recent guidelines do not maintain these differences and describe that protein intake of those training at higher frequency should fall in the highest end of the range [3]. Our data appear in agreement with this, because even though most of the amateur athletes we surveyed were not training at high frequency, we observed a positive correlation between protein intake and weekly training time.

In addition, we found that caloric distribution of macronutrients of amateur athletes are similar across sports types. Some studies evaluated protein dietary habits among elite athletes participating in different sport activities: in 2015, Baranauskas et al. [26] found that the protein intake was about 1.6 and 1.8 g/kg/day in a group of Lithuanian Olympic endurance athletes. Similarly, a study in Australian professional soccer players showed that protein intake was about 1.8 g/kg/day [27]. These studies suggest that athletes participating in sports relying on different metabolisms have similar protein intake. Moreover, amateur athletes tend to have lower protein intake; for example in a group of College athletes, a group more easily comparable with the one we evaluated in our survey with regard to training hours, a mean protein intake of 1.2 g/kg/day was observed [28].

We also did not find any notable gender-dependent difference in protein intake reported as grams per kilograms per day. There was no significant differences in training time between males and females when subdivided by sport group. In addition, protein intake was normalized to kilograms, which was the only data point that was significantly different between sexes. Our data suggest that our population consumes protein primarily based on weight (kg) and minimally based on hours of training, which is consistent with the guidelines.

When looking closer at the sub-group of athletes who did supplement their protein intake, an unexpected result was observed. Twenty-eight percent of the surveyed population supplemented their daily diet with protein supplements. Interestingly, when those who chose to supplement were separated from those who did not, the daily protein intake excluding intake from supplements was higher in those who chose to supplement, the exact opposite of what one would expect. One would presume that individuals who supplement their diet chose to do so because they are not meeting recommended values; however, these data suggest, that those who already have a sufficient level of daily intake with diet alone are the same individuals who also supplement their diet.

This type of pattern suggests that individuals who are aware of the importance of protein intake, and chose to supplement, are already making food consumption choices that would guarantee them an adequate amount of dietary intake.

Our survey shows, in addition, that in the subgroup not using protein supplements the percent of athlete below the suggested minimum range of 1.2 g/kg/day are high in aerobic, anaerobic and mixed, respectively, 14%, 48%, and 30%. Even considering that our data may not capture exactly the real nutritional status of these athletes due to the low accuracy of a three-day diary, these high values suggest that, aside from athletic coaching, nutritional coaching should be integrated into an athlete’s training living in Italy.

Overall, the Mediterranean diet appears as a dietary regimen able to provide sufficient protein to meet dietary recommendations in amateur athletes. A simple three-day food diary may be a feasible solution to raise awareness of an individual’s protein intake so to direct the necessary diet modification and/or suggest protein supplementation only when effectively needed.

## Figures and Tables

**Table 1:** Mean anthropometric measurements (weight, height, body mass index (BMI), and total training time of study participants).

		*n*	Weight (kg ± SD)	Height (m ± SD)	BMI (kg/m^2^ ± SD)	Training Time (hours/week ± SD)
**Aerobic**	total	40	66.6 ± 9.2	1.75 ± 0.07	21.7 ± 2.1	16.7 ± 8.1
	female	11	56.3 ± 5.6	1.68 ± 0.05	20.1 ± 1.9	18.8 ± 5.3
	male	29	70.4 ± 7.0	1.78 ± 0.06	22.2 ± 1.8	15.9 ± 8.8
**Anaerobic**	total	32	65.2 ± 12.1	1.71 ± 0.09	22.2 ± 2.7	6.7 ± 2.6
	female	15	55.9 ± 8.8	1.64 ± 0.08	20.7 ± 2.1	5.6 ± 1.7
	male	17	73.4 ± 7.8	1.77 ± 0.05	23.5 ± 2.4	7.7 ± 2.8
**Mixed**	total	94	74.5 ± 12.0	1.76 ± 0.08	23.9 ± 2.9	8.7 ± 11.1
	female	15	61.4 ± 9.2	1.65 ± 0.06	22.6 ± 3.0	9.7 ± 10.0
	male	79	77.0 ± 10.9	1.78 ± 0.06	24.1 ± 2.9	8.5 ± 11.2

**Table 2:** Summary of daily macronutrient caloric contribution according to sport type. Macronutrient intake (excluding supplement) is presented both as a percent of the total daily energy intake and as g/kg/day.

Summary of Macronutrient Consumption							
	*n*	Lipid % ± SD	Lipid g/kg/day ± SD	Carbohydrate % ± SD	Carbohydrate g/kg/day ± SD	Protein % ± SD	Protein g/kg/day ± SD
Total	166	29.8 ± 7.1	1.1 ± 0.4	47.6 ± 8.8	4.03± 1.3	17.5 ± 4.3	1.48 ± 0.46
Aerobic	40	29.4 ± 5.5	1.2 ± 0.4	47.9 ± 6.6	4.44 ± 1.2	17.7 ± 3.8	1.62 ± 0.43
Anaerobic	32	30.1 ± 9.7	1.0 ± 0.4	46.6 ± 9.0	3.64 ± 1.2	18.3 ± 4.7	1.41 ± 0.49
Mixed	94	29.8 ± 7.1	1.1 ± 0.4	47.6 ± 8.8	3.98 ± 1.4	17.5 ± 4.3	1.44 ± 0.45

**Table 3:** Mean of protein daily intake dividing the cohort among athletes who supplement with protein and athletes who do not.

	Does not Take Protein Supplementation Protein (g/kg/day ± SD)		Does Take Protein Supplementation Protein (g/kg/day ± SD)						
	***n***		***n***	**Without**			**With**		
**Aerobic**	21	1.46 ± 0.42	19	1.73	±	0.43	1.97	±	0.95
female	6	1.64 ± 0.38	5	1.90	±	0.33	2.18	±	0.50
male	15	1.48 ± 0.44	15	1.67	±	0.46	1.89	±	0.44
**Anaerobic**	23	1.24 ± 0.43	9	1.71	±	0.57	2.13	±	0.84
female	11	1.43 ± 0.45	3	1.39	±	0.63	2.37	±	0.71
male	12	1.17 ± 0.38	6	1.87	±	0.52	1.66	±	0.84
**Mixed**	76	1.41 ± 0.44	18	1.54	±	0.49	1.91	±	0.68
female	13	1.30 ± 0.50	2	1.62	±	0.21	1.78	±	0.37
male	63	1.44 ± 0.43	16	1.53	±	0.52	1.92	±	0.68

## Supplementary Materials

The following are available online at https://www.mdpi.com/2072-6643/12/12/3681/s1, Figure S1: Training time (hours/week) of athletes grouped by sport type and then by gender. Two-way ANOVA revealed significant sport type interaction (F (2, 160) = 45.73, *p* < 0.0001), as expected. However, the two-way ANOVA did not show a gender difference (F (1, 160) = 0.2753, *p* = 0.6005). Tukey’s post hoc multiple comparisons test showed that there was not gender difference between sexes in any sport group (n.s. = *p* > 0.05); Figure S2: (**a**) Percent of carbohydrates composing subjects’ daily caloric intake. (**b**) Percent of lipids composing subjects’ daily caloric intake. (**c**) Percent of protein composing subjects’ daily caloric intake. A two-way ANOVA was used to see if there were any sport or gender dependent variation on percent of macronutrients. There was no significant interaction detected by two-way ANOVA for percent carbohydrates (F (2, 160) = 0.5463, *p* = 0.5802) and percent lipids (F (2, 160) = 2.850, *p* = 0.0608). The percent protein intake was significant for an interaction, F (2, 160) = 8.304, *p* = 0.0004), and Tukey’s multiple comparisons test showed a significant difference between genders solely in the anaerobic sport group (*p* < 0.05); Figure S3: (**a**) daily protein intake (excluding supplements) subdivided by sport type and sex. Two-way ANOVA showed that protein intake did not vary by gender ((F (1, 160) = 1.378, *p* = 0.2422). Tukey’s test for multiple comparisons did not show any significant differences between gender in any sport type, n.s. = *p* > 0.05. (**b**) daily protein intake (excluding supplements) subdivided by sex of the entire population, Student’s t test did not show any significant differences n.s. = *p* > 0.05. Figure S4: Correlation between total protein intake (including supplements) and weekly training hours for athletes participating in different sport categories, (**a**) aerobic, (**b**) anaerobic, (**c**) mixed. Correlation analysis revealed a significant (*p* < 0.05) direct relation between the two variables. Table S1: Percent of population subdivided by sport and then by gender that consumed less than the minimum recommended daily protein intake of 1.2 g/kg/day.
