# Supplementary material for: The Mediterranean Athlete’s Nutrition: Are Protein Supplements Necessary?
Source: Nutrients. 2020 Nov 29;12(12):3681. doi: 10.3390/nu12123681 (PMC7759839; doi:10.3390/nu12123681)
Supplement: Supplementary file 1 [file nutrients-12-03681-s001.pdf]

nutrients

MDPI

Supplements

The Mediterranean Athlete's nutrition: are protein supplements necessary?

Catherine L. Passariello[1], Silvia Marchionni[2], Mariateresa Carcuro[3], Giorgia Casali[4], Alberto della Pasqua[5], Silvana Hrelia[6], Marco Malaguti[6]* and Antonello Lorenzini[2]

[1] Miami, FL, (USA);
[2] Department of Biomedical and Neuromotor Sciences, University of Bologna, Bologna (Italy);
[3] Castel San Pietro Terme, Bologna (Italy);
[4] Forlì, Forlì-Cesena (Italy)
[5] Poliambulatorio Obiettivo Benessere, San Mauro Pascoli, Forlì-Cesena (Italy);
[6] Department for Life Quality Studies, University of Bologna, Bologna (Italy);
* Correspondence: marco.malaguti@unibo.it

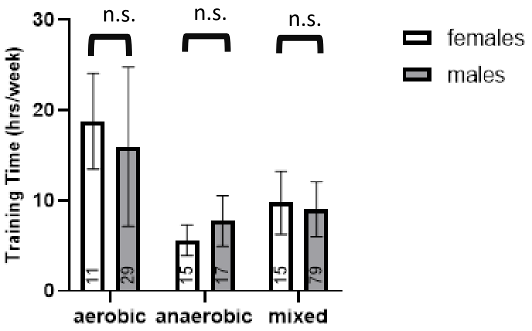

Supplemental Figure 1. Training time (hrs/week) of athletes grouped by sport type and then by gender. Two-way ANOVA revealed significant sport type interaction ($F(2, 160) = 45.73$, $P<0.0001$), as expected. However, the two-way ANOVA did not show a gender difference ($F(1, 160) = 0.2753$, $P=0.6005$). Tukey's post hoc multiple comparisons test showed that there was not gender difference between sexes in any sport group(n.s.=p>0.05).

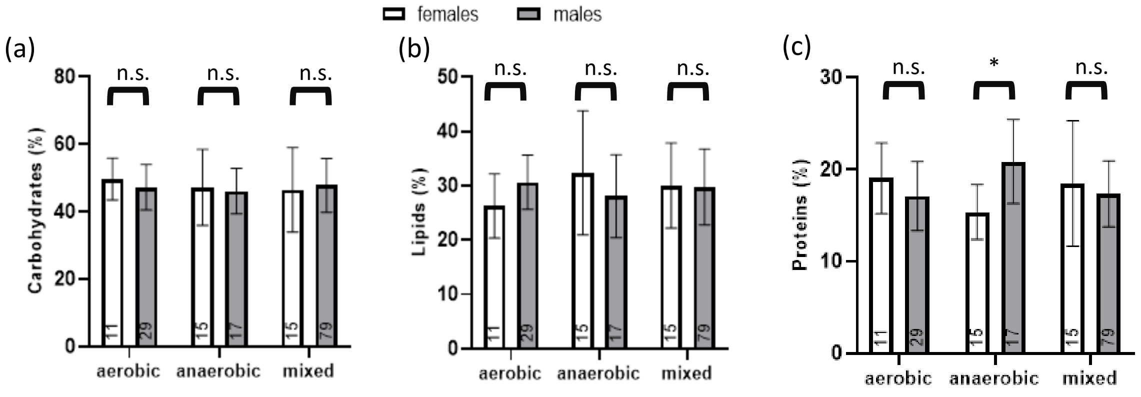

Supplemental Figure 2. (a) Percent of carbohydrates composing subjects' daily caloric intake. **(b)** Percent of lipids composing subjects' daily caloric intake. **(c)** Percent of protein composing subjects' daily caloric intake. A two-way ANOVA was used to see if there were any sport or gender dependent variation on percent of macronutrients. There was no significant interaction detected by two-way ANOVA for percent carbohydrates (F (2, 160) = 0.5463, P=0.5802) and percent lipids (F (2, 160) = 2.850, P=0.0608). The percent protein intake was significant for an interaction, F (2, 160) = 8.304, P=0.0004), and Tukey's multiple comparisons test showed a significant difference between genders solely in the anaerobic sport group (p<0.05)

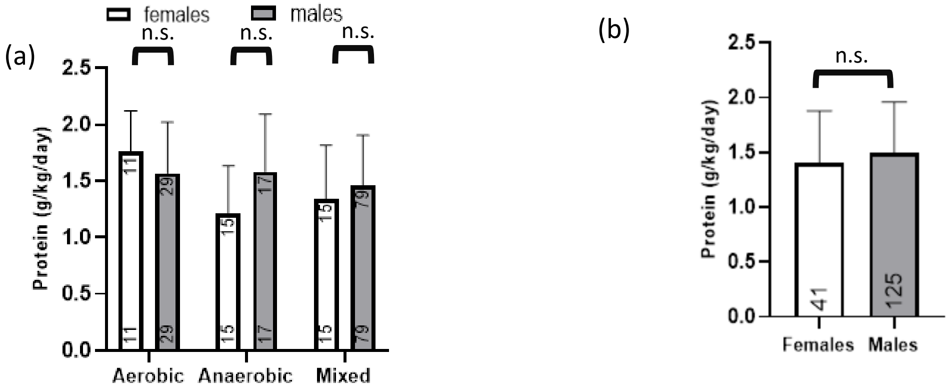

Supplemental Figure 3. (a) daily protein intake (excluding supplements) subdivided by sport type and sex. Two-way ANOVA showed that protein intake did not vary by gender ((F (1, 160) = 1.378, P=0.2422). Tukey's test for multiple comparisons did not show any significant differences between gender in any sport type, n.s.= p>0.05. (b) daily protein intake (excluding supplements) subdivided by sex of the entire population. Student's t test did not show any significant differences n.s.= p>0.05.

Supplemental Table 1. Percent of population subdivided by sport and then by gender that consumed less than the minimum recommended daily protein intake of 1.2 g/kg/day.

	**Does Not Take Protein Supplementation**		**Does Take Protein Supplementation**		
				**without**	**with**
	n	< 1.2 g/kg/day % (n)	n	< 1.2 g/kg/day % (n)	< 1.2 g/kg/day % (n)
**Aerobic**	21	14 (3)	19	5 (1)	5 (1)
**female**	6	0 (0)	5	0 (0)	0 (0)
**male**	15	20 (3)	15	7 (1)	7 (1)
**Anaerobic**	23	48 (11)	9	11 (1)	11 (1)
**female**	11	73 (8)	3	33 (1)	33 (1)
**male**	12	25 (3)	6	0 (0)	0 (0)
**Mixed**	76	30 (23)	18	28 (5)	11 (2)
**female**	13	46 (6)	2	0 (0)	0 (0)
**male**	63	27% (17)	16	31 (5)	13 (2)

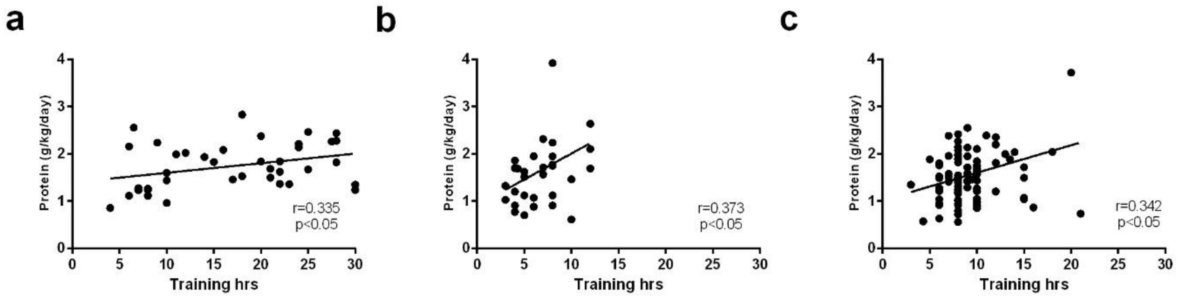

Supplemental Figure 4. Correlation between total protein intake (including supplements) and weekly training hours for athletes participating in different sport categories, (a) aerobic, (b) anaerobic, (c) mixed. Correlation analysis revealed a significant ($p<0.05$) direct relation between the two variables.

Three Days Dietary Journal

Gender:

Age:

Weight (kg):

Haight (m):

INSTRUCTIONS FOR FILLING IN THE DIARY:

The diary has to be completed describing the type of foods consumed and their quantity. The quantities can be described using weight, universal measures (spoon etc. ...), or portions given by food packaging.

All foods, liquid or solid, eaten during the day and therefore also snacks (including sweets, gum etc ..) must be recorded.

When the composition of a food preparation is not standard, indicate the preparation's name and the quantity eaten and attach a brief description of the recipe or ingredients.

For each meal, the following must be indicated:

- Eating hours

- Place of eating

- type of FOOD specifically (eg skimmed milk, whole milk, partially… specify) and, if present, the brand of food. Important: the DRESSINGS (oil, butter, margarine ... and so on) and any SUGAR or SWEETENER that are also included in the food table

- Quantity eaten

At the end of each recorded day, indicate if you took protein, vitamin or micronutrient supplements, the quantity, brand and time of intake.

If you consume foods such as snacks, supplements, in addition to indicating the quantity and brand in the diary, remember to attach a photo of the food composition tables that are generally found on the back / edge of the package.

Day x: (please fill the diary for every days)

Date:

Week day:

Sport Training (Describe the activity you perform and how long it lasts):

Breackfast:

Time:

Where did you eat it?:

FOODS (include dressing)	BRAND (If any)	AMOUNT

Notes:

Morning SNACK:

Time:

Where did you eat it?:

FOODS (include dressing)	BRAND (If any)	AMOUNT

Notes:

LUNCH:

Time:

Where did you eat it?:

FOODS (include dressing)	BRAND (If any)	AMOUNT

Notes:

Afternoon SNACK:

Time:

Where did you eat it?:

FOODS (include dressing)	BRAND (If any)	AMOUNT

Notes:

DINNER:

Time:

Where did you eat it?:

FOODS (include dressing)	BRAND (If any)	AMOUNT

Notes:

DRINKS consumed in the day

DRINK	BRAND (If any)	AMMOUNT

DIETARY SUPPLEMENTS (list Name, Brand and Ammount of any supplement you took in the day):
